# Effects of rehabilitation therapy based on exercise prescription on motor function and complications after hip fracture surgery in elderly patients

**DOI:** 10.1186/s12891-023-06806-y

**Published:** 2023-10-14

**Authors:** Yan-Jun Che, Zongna Qian, Qi Chen, Rui Chang, Xiaofeng Xie, Yue Feng Hao

**Affiliations:** 1 Orthopedics and Sports Medicine Center, Affiliated Suzhou Hospital of Nanjing Medical University, Suzhou, Jiangsu Province 215008 China; 2 Department of Rehabilitation Sciences, The Hong Kong Polytechnic University, Hong Kong SAR, 999077 China

**Keywords:** Hip fracture, Daily living ability, Exercise prescription, Rehabilitation, Motor function, Postoperative complications, Elderly patients

## Abstract

**Background:**

Exercise rehabilitation training is an important measure for improving the prognosis of patients with hip fractures. However, the particular program that works effectively and the efficiency of exercise therapy are still controversial.

**Objective:**

To compare the effects of usual postoperative care combined with rehabilitation based on exercise prescription on motor function and complications in elderly patients who underwent surgery for hip fracture.

**Methods:**

This was an observational study. A total of 71 elderly patients with hip fractures who were treated with hip arthroplasty and internal fixation of the proximal femur with an intramedullary nail at Suzhou Municipal Hospital from October 2020 to December 2021 were included; 11 cases were excluded (eight cases were excluded due to loss of follow-up, two due to deaths from other causes, and one due to other reasons). Finally, 60 patients (18 males and 42 females) were included. Patients were randomly assigned to the control (n = 30) and experimental (n = 30) groups using a random number generator. Patients in the control group received usual postoperative care, whereas those in the experimental group received usual postoperative care combined with rehabilitation training based on the principles of exercise prescription. We recorded the motor function (Harris hip score), daily living ability (Barthel Index), and complications at discharge and 1, 3, and 6 months postoperatively for statistical analysis.

**Results:**

The Harris hip score and Barthel Index score were significantly higher at 1, 3, and 6 months postoperatively than at discharge in both groups (p < 0.05). The Harris hip score and Barthel Index score at discharge and 1, 3, and 6 months postoperatively were significantly higher in the experimental group than in the control group (p < 0.05). The incidence of complications at 6 months postoperatively was significantly lower in the experimental group than in the control group (13% vs. 37%).

**Conclusions:**

Rehabilitation therapy based on exercise prescription helps improve hip function and the ability to perform activities of daily living and related postoperative complications after hip fracture surgery in elderly patients. The findings of our study will guide decision-making in clinical practice and improve the clinical management of hip fractures in elderly patients postoperatively.

## Introduction

Osteoporotic fractures, particularly hip fractures, are one of the leading causes of disability and death in elderly patients [1–4]. Compared with patients without a history of fracture, patients with a primary fracture have twice the risk of a second fracture, with a fatality rate of 34.8% [4]. The incidence of hip fractures due to osteoporosis is predicted to increase globally in the coming decades, along with an increase in medical care and social security costs [5–7]. Inadequate early treatment is strongly associated with a decreased quality of life and functional deficits in the future in elderly patients with hip fractures [8]. Surgery and rehabilitation are required in almost all patients after fractures. Furthermore, surgery within 48 h after a patient’s admission can significantly reduce mortality [9, 10]. The international guidelines for the management of patients with hip fractures recommend that patients must undergo rehabilitation training from day 1 postoperatively unless they have contraindications [11, 12]. Rehabilitation training is an important factor in functional recovery and improvement of the quality of life of patients [13, 14] and exerts a positive effect by preventing the deterioration of daily function and promoting the recovery of motor function [14–17]. Furthermore, rehabilitation training should be progressive, individualized, and cyclical for improving the prognosis of patients with hip fractures [15]. However, there is a lack of detailed programs on exercise therapy for the treatment of postoperative patients with hip fractures. In this study, we designed a progressive and scientific exercise therapy program based on the research advances and clinical guidelines on the rehabilitation of patients after hip fracture surgery [11, 12] in combination with the concept of exercise prescription and also assessed its usefulness in patients after hip fracture surgery.

## Methods

### General data

This was an observational study. The study was approved by the Ethics Committee of the Affiliated Suzhou Hospital of Nanjing Medical University (Suzhou Municipal Hospital); Informed consent was obtained from all the participants. A total of 71 elderly patients with hip fractures who were treated with hip arthroplasty and internal fixation of the proximal femur with an intramedullary nail at Suzhou Municipal Hospital from October 2020 to December 2021 were included; 11 cases were excluded (eight cases were excluded due to loss of follow-up, two due to deaths from other causes, and one due to other reasons). Finally, 60 patients (18 males and 42 females) were included. The patients were randomly assigned to the control (n = 30) and experimental (n = 30) groups using a random number generator.

The inclusion criteria were as follows: (1) patients diagnosed with femoral neck or intertrochanteric fracture confirmed by the physician’s subjective and objective examination and imaging data findings (anteroposterior and lateral X-ray and computed tomography examination of pelvis or hip joint) and those who underwent relevant surgery (total or half hip arthroplasty and internal fixation with proximal femoral intramedullary nail); (2) patients willing to participate in the study; (3) patients aged ≥ 65 years [18]; (4) patients who were able to perform daily living activities before fracture (Barthel Index > 60) [19].

The exclusion criteria were as follows: (1) patients with cognitive impairment and inability to undergo rehabilitation training (Simple Mental State Examination score < 27); (2) patients with a combination of other joint musculoskeletal disorders; or (3) patients with a combination of other inflammatory or autoimmune diseases.

We assessed the postoperative hip function at discharge and 1, 3, and 6 months postoperatively. The endpoints/outcomes of the study were fractures after surgery or death.

### Surgery

Patients in both groups underwent surgical treatment as soon as possible after the diagnosis of fracture; the surgeon used different operation modes depending on the type of injury. The main procedures performed were hip arthroplasty and internal fixation of the proximal femur with an intramedullary nail. All surgeries were performed by the same group of surgeons at the Department of Orthopaedics, Suzhou Municipal Hospital. Among the 30 patients in the control group, 12 underwent hip arthroplasty and 18 underwent internal fixation of the proximal femur with intramedullary nails. Among the 30 patients in the experimental group, 10 underwent hip arthroplasty and 20 underwent internal fixation of the proximal femur with intramedullary nails. All patients underwent surgeries without any unexpected events.

### Usual postoperative care

Patients in both groups received the usual postoperative care. All nursing staff received uniform training before the initiation of the experiment and conducted nursing interventions on patients according to a standard procedure.

The main contents of the nursing interventions were as follows: (1) multimodal analgesia (oral medication or intravenous self-controlled analgesic pump as required); (2) postural care: good posture placement and regular turn-over to prevention; (3) monitoring of postoperative vital signs and providing anti-infection treatment; (4) lifestyle management: recording the time of second stool excretion, diet plan, routine guidance, and education on sleep and psychology.

### Rehabilitation program based on exercise prescription

The basic elements of the exercise prescription included Frequency, Intensity, Time, Type, Volume, and Progression, (FITT-VP) [20]. Accordingly, 30 patients underwent a systematic and individualized training program preoperatively, postoperatively, and after discharge, respectively.

1. The main aim of preoperative exercise therapy is to maintain the cardiopulmonary capacity of the patients and prevent complications. The main exercises were moderate-intensity upper limb resistance training, respiratory training, ankle pump exercise, and lower limb isometric contraction training. Each training lasted for 15–30 min and was done every day preoperatively.
2. The main aim of postoperative exercise therapy is to improve hip joint mobility and the muscle strength of the lower limbs. Weight-transfer training was conducted with the permission of the physician. The primary exercises were low-to-moderate intensity hip flexibility training, strength training, and balance coordination training, which were performed for 30 min per session and 5 times every week.
3. The main aim of exercise therapy after discharge is to improve the ability of the patient to perform activities of daily living and continue to support the balance and coordination training of the lower limbs to decrease the onset of falls. Daily living training and balance training were conducted step by step with low-to-moderate intensity for 15–30 min each time and 3–5 times every week. The details are as follows:

**Table:** 

Preoperative	1. Abdominal respiration: lie down or in a semi-recumbent position, inhale through the nose, and exhale through the mouth. The upper abdomen should bulge out when breathing in, and the abdomen should retract when breathing out. Breathing should be slow, fine, and even, 5–10 min/time, 2–5 times/day 2. Abdominal massage: in the supine position, place the base of the palm on the abdomen and slowly and gently massage in a clockwise direction, 5–10 min/time, 2–5 times/day 3. Ankle pump exercise: forceful, slow, full range of flexion and extension of the ankle joint, hook the foot to the end for 5 s and then tense the foot for 5 s, 10 sets/group, 1–3 sets/h 4. Static contraction of quadriceps femoris: straighten the knee joint on the affected side, tighten anterior thigh muscles for 10 s, and then relax, 10 pieces/group, 2 or 3 groups/time, 2 times/day 5. Single bridge exercise: in the supine position, bend the hip and knee to 90 degrees on the healthy side, raise the affected side and tighten and raise the hip, 10 times/group, 3?5 s between each time, 1 or 2 groups/day 6. Double upper limb resistance group training: a. In the supine position or end sitting position, hold 0.5?2 kg dumbbells with both hands to do front and side lifting exercises. b. Hold 10–15 kg elastic band with both hands to do resistance group chest expansion exercise. c. Pull the rings with both hands, tighten the abdominal muscles, and raise the torso. The movements are required to be slow and even, 5–10 min/time, 2 times/day Treatment can be combined with instruments: pneumatic, low-to-moderate frequency, magnetic therapy, infrared, etc. (use as appropriate)
Postoperative	Continue to use the above exercises and add the following as appropriate: 1. Strength exercises: strength training of the peripatellar muscle groups: do forward flexion, abduction, and back extension training of the hip joint in different positions, from passive—assisted—fully active. Note that the hip must not be internally rotated, hold for 5–10 s at the end of the activity, 10 sets/group, 2 or 3 groups/time, 2 times/day 2. Balance training: a. Sitting balance training: according to the patient’s condition, perform sitting three-stage balance training with or without weight-bearing on the affected limb, 2–5 times/repetition, 2–3 times/day, and b. Standing balance training: according to the patient’s condition, perform progressive weight-bearing exercises on the affected limb using the assistive device, 2–5 times/repetition, 2–3 times/day 3. Walking training with assistive devices: walking training with a walker: note that when turning around, you should first take a step to the side of the turn, move the walker, and then follow the other limb, 50–100 m/repetition, 2–3 times/day Treatment can be combined with instruments: pneumatic, low-to-moderate frequency, magnetic therapy, infrared, etc. (use as appropriate)
Out of Hospital	Selecting the above exercises, you can add the following: 1. Walking training (10–15 min/time, 2 times/day): walking training with a walker: note that when turning around, you should first take a step to the side of the turn, move the walker, and then follow up with the other limb. If the patient is holding a double crutch, use four-point step training, 50–100 m/time, 2–3 times/day 2. Daily living ability training (10–15 min/time, 2 times/day): a. Stair climbing training: climb up the stairs in the order of healthy side—affected side—abduction and walk down the stairs in the order of affected side—abduction—healthy side; b. Transfer training; and c. Alternate positions when putting on and taking off trousers, putting on and taking off shoes and socks, and picking up objects on the floor
Notes	1. Perform low-to-moderate intensity training as the main focus and progress gradually 2. Avoid holding your breath and combine the exercises with breathing exercises (abdominal breathing) 3. After each exercise, apply cold compresses immediately for 10–15 min each time 4. If the following conditions occur, the exercise should be stopped immediately: a. Feeling chest pain, difficulty in breathing, dizziness, nausea, and vomiting during exercise b. Having a blood pressure of > 200/100 mmHg or a systolic blood pressure increase of > 30 mmHg or a decrease of 10 mmHg during exercise c. During exercise, the ST segment monitored by the electrocardiogram is shifted downward by ≥ 0.1 mV or increases by ≥ 0.2 mV 5. After hip arthroplasty: a. if the posterior-lateral approach is used, avoid flexion of the hip beyond 90°, excessive rotation, and adduction; b. if the anterolateral approach is used, avoid hyperextension and external rotation of the hip

### Assessment index

**Harris Hip Function Scoring Scale** [21]: This scale is used to evaluate four dimensions including pain, function, activity, and hip joint mobility; the scale score ranges from 0 to 100: 90–100 is excellent, 80–89 is good, 70–79 is fair, and < 70 is poor.

**Barthel Index** [19]: This index is sued for assessing the ability to perform activities of daily living and uses a total of 10 dimensions with a total score of 100: 100 is considered normal, 61–99 shows slight dependence; 41–60 is moderately dependent; and ≤ 40 shows heavy dependence.

**Complications**: The frequency of complications was recorded for both groups 6 months postoperatively in four areas: pressure sores, lower limb muscle atrophy, deep vein thrombosis, and reduced cardiopulmonary function.

### Statistical methods

Data from this study were statistically analyzed using SPSS version 23.0 (IBM SPSS Inc., Chicago, IL, USA). The Shapiro–Wilk test was used to test the normal distribution of the functional questionnaire scores of all patients, and exploratory analysis was used to calculate subject demographics. An independent sample t-test was used to evaluate the function between the two groups; the chi-square test was used to compare the difference in complications between the two groups within 6 months; We set α as 0.05, efficacy 1-β as 0.8, OR = 4, and obtained a sample size of at least 30 people in each group (n = 30). All statistical methods were tested at a level of alpha of 0.05. Count data were expressed as cases and percentages; measurement data were expressed as mean ± standard deviation.

## Results

### Demographic data

The experimental group included 10 males and 20 females (mean age: 81.8 ± 6.1 years); the control group included 8 males and 22 females (mean age: 82.5 ± 4.5 years). No significant differences were observed in the indexes between the two groups at baseline (p > 0.05; Table 1).

**Table 1:** Baseline characteristics of participants with hip fracture (n = 60)

Group	Age	Sex (%)		Mode of surgery (%)	
		Man	Female	Hip replacement	Internal fixation
Control (n = 30)	82.5 ± 4.5	8 (27%)	22 (73%)	12 (40%)	18 (60%)
Experimental (n = 30)	81.8 ± 6.1	10(33%)	20 (67%)	10 (33%)	20 (67%)
p	0.629	0.547		0.771	

### Comparison of harris hip scores

The Harris hip scores at 1, 3, and 6 months postoperatively were significantly higher than those at discharge in both groups (p < 0.05); the Harris hip scores at discharge and 1, 3, and 6 months postoperatively were significantly higher in the experimental group than in the control group during the same period (p < 0.05; Table 2).

**Table 2:** Comparison of Harris hip scores

Group	Discharge	1 months post-op	3 months post-op	6 months post-op
Control (n = 30)	45.11 ± 6.09	62.17 ± 6.24	76.14 ± 7.55	78.88 ± 10.67
Experimental (n = 30)	56.33 ± 7.41	70.82 ± 6.73	83.32 ± 8.39	88.48 ± 9.61
p	0.043	0.039	0.031	0.037

Post-op: postoperatively

### Comparison of barthel index

The Barthel Index scores at 1, 3, and 6 months postoperatively were significantly higher in both groups than in this group at discharge (p < 0.05); the Barthel Index scores at discharge and 1, 3, and 6 months postoperatively were significantly higher in the experimental group than in the control group at the same time (p < 0.05; Table 3).

**Table 3:** Comparison of Barthel Index

Group	Discharge	1 months post-op	3 months post-op	6 months post-op
Control (n = 30)	44.19 ± 7.22	65.00 ± 8.27	74.61 ± 8.69	75.35 ± 8.75
Experimental (n = 30)	56.27 ± 7.48	72.35 ± 8.43	80.38 ± 8.15	89.05 ± 10.80
p	0.046	0.039	0.041	0.036

Post-op: postoperatively

### Comparison of the occurrence of complications within 6 months

A total of 11 complications occurred in the control group (pressure sores = 2; muscle atrophy of the lower limbs = 4; reduced cardiopulmonary function = 5); however, only 4 complications occurred in the experimental group (muscle atrophy of the lower limbs = 2; reduced cardiopulmonary function = 2). No deep vein thrombosis was noted in either group. In terms of overall incidence, the probability of complications within 3 months postoperatively was significantly lower in the experimental group (13%) than in the control group (37%) (Table 4).

**Table 4:** Comparison of the occurrence of complications within 6 months (%)

Group	PS	MA	DVT	RAF	OI
Control (n = 30)	2 (0.07)	4 (0.13)	0 (0.00)	5 (0.17)	0.37
Experimental (n = 30)	0 (0.00)	2 (0.066)	0 (0.00)	2 (0.066)	0.13

PS, pressure sores; MA, muscle atrophy; DVT, deep vein thrombosis;

RAF, reduced cardiopulmonary function; OI, overall incidence

## Discussion

Hip fracture is one of the most common injuries with the worst prognosis in the elderly; its incidence, mortality, and treatment costs have attracted widespread attention from clinicians [1, 2, 22, 23]. Progressive, individualized, and periodic postoperative rehabilitation is an essential measure to restore the function of the affected limb and improve the quality of life of the elderly [13, 15, 24]. The present study compared the effects of usual postoperative care and rehabilitation treatment based on the principles of exercise prescription (FITT-VP) on the hip function and quality of daily life of elderly patients after hip fracture surgery. Our study findings revealed that rehabilitation training based on exercise prescription has considerable advantages in improving hip function, increasing the ability to perform activities of daily living, and decreasing the incidence of related complications after hip fracture surgery in the elderly. These results have important guiding significance for the postoperative clinical management of elderly patients with hip fractures.

Elderly patients with hip fractures have a significantly reduced ability to perform activities of daily living and use tools compared with their pre-injury status [25]. Furthermore, only 25% of patients can return to their pre-injury functional status, > 56% are left with severe disability, 50% require mobility aids, and only 53.9% can walk independently [26]. Moreover, 86.2% of patients who underwent rehabilitation at an early stage were able to successfully return to life in society [27]. Rehabilitation programs after hip fracture surgery can be effective in improving hip function, the ability to perform activities of daily living, pain, and mortality in patients [27–29]. However, the clinical benefits of different exercise treatment interventions are not the same as they depend on the type, frequency, and intensity of the rehabilitation exercise and the differences in the fitness level of the patient.

In the elderly, hip fracture is a localized bone tissue lesion based on the presence of systemic osteoporosis, a clear manifestation of reduced bone strength in the hip, and a final consequence of osteoporosis. Hip fracture in the elderly is directly caused by falling and is usually affected by the interaction of multiple risk factors, including environmental, physiological, and neuromuscular diseases [30]. Elderly patients often experience a second fracture of the opposite hip soon after surgery of one hip (usually within 1 year), causing great economic loss and psychological and emotional damage to society, families, and the patients themselves. Compared with patients with no history of fracture, elderly patients with osteoporotic fractures are twice as likely to suffer a second fracture, with the risk of a second fracture being highest within the first year, with a mortality rate of 34.8% [4]. There are several reasons for this: first, the patients themselves are not aware enough of the postoperative risks of fractures and assume that if they do not move, they will not fall and end up having another fracture after fracture surgery. This happens because the patients are unaware that immobility or lack of movement will lead to further loss of bone mass, reduced muscle strength, and imbalance, which will more easily exacerbate the existing osteoporosis and make it easier to fall again. Second, the patients themselves along with their families and even some doctors do not pay enough attention to the root cause of hip fractures in the elderly, i.e., osteoporosis and the subsequent imbalance of skeletal and muscular strength. The hip is an attachment site for many of the core body muscles and also the site of posture maintenance and stress resistance. Hip fractures disturb the balance and coordination of the adjacent muscle groups and there is decreased skeletal stress stimulation caused by forced bed rest after the fracture; therefore, a vicious cycle of osteoporosis of the hip joint is started. Third, muscle strength is important for maintaining normal function in the elderly. Furthermore, decreased muscle strength is a risk factor for repeated falls and hip fractures [31] Leg muscle strength continues to reduce at a rate of 1–2% in older people beyond the age of 65 years, indicating that older people are at risk of losing mobility and have an increased risk of falls [32]. Furthermore, reduced exposure to the sun can significantly increase the risk of hip fractures [33, 34]. Vitamin D promotes the absorption and metabolism of calcium and phosphorus and is also an important factor that maintains muscle coordination. The blood levels of vitamin D drop in winter particularly due to less exposure to the sun as the skin is usually covered. Therefore, by providing vitamin D supplements and increasing the time to sun exposure, calcium can be effectively absorbed and the risk of falls can be reduced [35]. Thus, a suitable living environment is an important factor for improving postoperative prognosis and exercise adherence in elderly patients.

Exercise prescription is a new rehabilitation treatment mode for sequential recovery of hip fractures in the elderly. One-third of hip fractures can be prevented by performing regular physical activity [36]; regular exercise can reduce the risk of hip fracture by 31% and that of arbitrary fracture by 19% [37]. The rehabilitation treatment program used in our study was based on the principles of exercise prescription, which mainly focused on preoperative moderate-intensity upper limb resistance training, respiratory training, ankle pump exercise, and lower limb isometric contraction to maintain the cardiopulmonary function of the patients and prevent bed-ridden complications. Low-to-moderate intensity hip flexibility training, strength training, and balance coordination training were provided postoperatively, with the main aim of improving hip mobility and lowering limb muscle atrophy; weight-transfer training was conducted with the permission of the physician. After discharge from the hospital, daily living training and balance training were progressively conducted mainly with low-to-moderate intensity exercises. The main aim was to improve the patients’ ability to perform activities of daily living and to continue to strengthen the balance and coordination of the lower limbs, thus decreasing the incidence of falls. The rehabilitation therapy based on exercise prescription in our study had an adequate effect on improving the hip function and daily living ability of elderly patients. This may be because the usual standardized and systematic training increases muscle function, joint mobility, and cardiopulmonary capacity; on the other hand, progressive rehabilitation additionally improves the patients’ fear and helps them regain their confidence in rehabilitation. Moreover, patients who regularly exercise postoperatively can actively go outdoors to receive sunlight, which is one of the main ways to actively increase bone density and reduce hip fractures.

Postoperative complications are effective indices for assessing the quality of care and rehabilitation, which are also closely related to decreased postoperative disability and mortality rates [38]. The results of our study suggest that rehabilitation based on exercise prescription is effective in reducing the possibility of postoperative complications, particularly in terms of muscle atrophy and cardiopulmonary function. Furthermore, Tedesco et al. noted that patients who underwent postoperative rehabilitation treatment at the hospital had a lesser mortality rate than those who did not receive postoperative rehabilitation [17]. Therefore, it is important to use rehabilitation training based on exercise prescription as a usual method of treatment for the management of patients with hip fractures. However, it is unusual that some patients who underwent rehabilitation training still developed lower limb muscle atrophy and decreased cardiopulmonary function. This may be because patients in this age group have more pre-fracture co-morbidities and there is a lack of supervision of post-discharge rehabilitation training and compliance with the training program, suggesting that clinical staff need to strengthen out-of-hospital follow-up and supervision. In particular, professional cooperation and multidisciplinary management among geriatricians, orthopedic surgeons, anesthetists, physiatrists, physiotherapists, and general practitioners should be strengthened to guide patients to undergo progressive rehabilitation training in and out of the hospital [39].

Nevertheless, our study has certain limitations. First, all patients underwent either hip replacement (hemi- or total hip) or intramedullary nail fixation. Although a rigorous experimental design was used to randomly assign patients to experimental and control groups to minimize the differences at baseline, there might be a difference in the specific requirements for rehabilitation between the two different surgical modes; therefore, future studies need to further refine the rehabilitation program in different subgroups. Second, our study had a small sample size and a short follow-up duration. We included only 30 patients in each group, and the patients were followed up for only 6 months. Moreover, patients with hip fractures have significantly reduced functional capacity 1 year after discharge from the hospital after receiving rehabilitation; more than half of the elderly patients with hip fractures are unable to maintain the effect of postoperative rehabilitation 1 year after discharge [40]. Therefore, future studies with a larger sample size and long-term rehabilitation program design are required to observe the long-term therapeutic effects of rehabilitation training based on exercise prescription.

In summary, the main aim of rehabilitation after hip fracture in the elderly is to successfully discharge the patients from the hospital and help them return to pre-fracture functional status, thus decreasing the health and financial burden of poor prognosis. Rehabilitation program based on the principles of exercise prescription has strong advantages in the postoperative management of patients with hip fractures, which can in turn effectively improve their hip function and ability to perform activities of daily living. Therefore, clinicians need to be aware of the requirement to provide individualized and cyclical rehabilitation therapy based on exercise prescription to elderly patients and strengthen the supervision of such patients.

## Data Availability

The datasets used and/or analysed during the current study available from the corresponding author on reasonable request.
